# Exposure to deltamethrin affects development of *Plasmodium falciparum* inside wild pyrethroid resistant *Anopheles gambiae* s.s. mosquitoes in Uganda

**DOI:** 10.1186/s13071-016-1384-x

**Published:** 2016-02-24

**Authors:** Mojca Kristan, Jo Lines, Anthony Nuwa, Charles Ntege, Sylvia R. Meek, Tarekegn A. Abeku

**Affiliations:** Department of Disease Control, London School of Hygiene & Tropical Medicine, London, UK; Malaria Consortium Uganda, Kampala, Uganda; Kyankwanzi District Health Office, Ministry of Health, Butemba, Uganda; Malaria Consortium, London, UK

**Keywords:** Malaria, *Anopheles gambiae*, Insecticide resistance, Pyrethroids, *Plasmodium falciparum*, Oocyst, Sporogony

## Abstract

**Background:**

Pyrethroid resistance in African vector mosquitoes is a threat to malaria control. Resistant mosquitoes can survive insecticide doses that would normally be lethal. We studied effects of such doses on *Plasmodium falciparum* development inside *kdr*-resistant *Anopheles gambiae* s.s. in Uganda.

**Methods:**

We collected *An. gambiae* s.s. homozygous for *kdr*-L1014S mutation, fed them on blood samples from 42 *P. falciparum*-infected local patients, then exposed them either to nets treated with sub-lethal doses of deltamethrin or to untreated nets. After seven days, we dissected 692 mosquitoes and examined their midguts for oocysts. Prevalence (proportion infected) and intensity of infection (number of oocysts per infected mosquito) were recorded for each group.

**Results:**

Both prevalence and intensity of infection were significantly reduced in deltamethrin-exposed mosquitoes, compared to those exposed to untreated nets. With low doses (2.5–5.0 mg/m^2^), prevalence was reduced by 59 % (95 % CI = 22 %-78 %) and intensity by 41 % (95 % CI = 25 %-54 %). With high doses (10–16.7 mg/m^2^), prevalence was reduced by 80 % (95 % CI = 67 %-88 %) and intensity by 34 % (95 % CI = 20 %-46 %).

**Conclusions:**

We showed that, with locally-sampled parasites and mosquitoes, doses of pyrethroids that are sub-lethal for resistant mosquitoes can interfere with parasite development inside mosquitoes. This mechanism could enable pyrethroid-treated nets to prevent malaria transmission despite increasing vector resistance.

## Background

Increased use of insecticide-treated nets (ITNs) has contributed to substantial reductions in the global burden of malaria [1]. Unfortunately, various genes conferring resistance to pyrethroids are spreading rapidly through the main African malaria vectors [2, 3]. However, the impact of this resistance on vector control remains unclear [4]. Control failure has been associated with resistance in some areas [5], but not others [6–8].

Pyrethroid-treated nets reduce malaria transmission partly by repelling vectors, and partly by killing them [9]. As resistance increases, the proportion of the vector population surviving insecticide exposure increases. Although this is expected to reduce the effectiveness of vector control, it is possible that transmission might still be prevented by other mechanisms. One possible mechanism is that infection might restore the phenotypic susceptibility of genetically resistant mosquitoes, so they are killed by doses that they would survive without the infection. Another is through a possible effect of the insecticide on the parasite. With increasing resistance, the proportion of the vector population exposed to sub-lethal doses is also expected to increase, which in turn increases exposure of the parasite to the insecticide. Exposure of the parasite to these doses inside the mosquito might affect its development even though the insecticide fails to kill the mosquito. Either of these mechanisms could, in theory, allow insecticide resistance to evolve in a vector population with little impact on malaria transmission.

A number of studies have investigated potential effects of insecticides and insecticide resistance on parasite development. Resistant mosquitoes infected with *Plasmodium falciparum* have been found to be more susceptible to DDT than uninfected mosquitoes [10]. Other studies reported that *Anopheles gambiae* with knock-down resistance (*kdr*) genes exhibited increased susceptibility to *P. falciparum* [11, 12].

In one study, exposure to DDT and bendiocarb inhibited development of *P. falciparum* in insecticide-resistant *An. gambiae* s.s. [13]. Sub-lethal doses of pyrethroids were also shown to affect development of *Plasmodium* parasites in laboratory conditions [14–16]. However, other studies found no effect of organochlorines, carbamates and organophosphates on parasite development in mosquitoes [17–19].

The late Nigel Hill carried out laboratory-based research at London School of Hygiene & Tropical Medicine (LSHTM) on the effects of sub-lethal doses of pyrethroids on *Plasmodium* infection rates in *An. stephensi* mosquitoes [17]. He infected pyrethroid susceptible and resistant mosquitoes with the rodent parasite *P. yoelii nigeriensis*. Mosquitoes were exposed to deltamethrin-, permethrin- and lambda-cyhalothrin-treated nets and papers. Exposure to these pyrethroids before, during or after infective feed significantly reduced prevalence of infection under laboratory conditions. He subsequently carried out similar laboratory-based experiments using a pyrethroid resistant *An. stephensi* strain and a laboratory strain of *P. falciparum.* Again, exposure to permethrin shortly after infective feed caused a significant reduction in the infection prevalence.

Our aim was therefore to demonstrate that this phenomenon can occur in the field. We investigated the effects of deltamethrin exposure of wild, pyrethroid-resistant *An. gambiae* s.s. on the sporogonic development of *P. falciparum* parasites obtained from local patients at a health facility in a malaria endemic area of Uganda. The range of insecticide exposures was selected to resemble those that blood-seeking mosquitoes might be expected to encounter in an area where the nets are not new, and where the concentration of insecticide on nets is considerably lower than in new nets.

## Methods

### Study area and participants

The study was conducted in Butemba, Kyankwanzi District, mid-western Uganda, between August 2013 and June 2014. Butemba (approximately 200 km north-west of Kampala) lies at an altitude of 1000–1200 m above sea level in a moist savannah zone, with annual rainfall exceeding 1200 mm with two peaks (April–May and September–October). The study site included the catchment area of Butemba Health Centre III, which is mostly rural but includes a semi-urban village of Bukwiri. The area is highly endemic with two peaks in malaria transmission in May–July and October–December.

Forty-two gametocyte donors were recruited among outpatients at Butemba Health Centre III. Patients who fulfilled the inclusion criteria (2 years or older, *P. falciparum* positive with microscopically detectable gametocytes, no sign of severe illness, non-pregnant if adult female, and haemoglobin level of >9.9 g/dl) were recruited. Gametocytes were counted against 200 leucocytes in thick blood smears. Density was calculated assuming a standard leukocyte count of 8000/μL of blood. The experiments were conducted over three rounds (September–October 2013, November–December 2013, and May–June 2014).

### Mosquito collection and rearing

*Anopheles gambiae* s.l. larvae were collected from breeding sites in villages around the health centre and reared at the health centre at ambient temperature and humidity. The emerging adult mosquitoes were given 10 % glucose solution until they were fed on infected blood.

### Mosquito species and resistance studies

World Health Organization (WHO) susceptibility tests were conducted using different classes of insecticides to assess the phenotypic resistance levels in the study area [20]. All mosquitoes used in the transmission experiments and the WHO susceptibility tests were stored dry on silica gel for molecular analysis. Real-time polymerase chain reaction (qPCR) using TaqMan assays was used for *Anopheles* sibling species identification [21], and for detection of *kdr-*L1014F or *kdr-*L1014S mutations [22]. A further assay to detect the presence of G119S mutation in the gene *ace-1* which encodes the acetylcholinesterase enzyme was also used [23].

### Experimental nets

Untreated polyester nets (Vestergaard) were treated with a range of concentrations (2.5–16.7 mg/m^2^) of deltamethrin (K-Othrine SC 10B G, concentration 9.7 g/l; Bayer CropScience AG). The doses were much lower than those used on LLINs, and were chosen in an attempt to mimic the concentrations found on nets as they get older in domestic use [24].

### Procedures

Approximately 9 ml blood was collected from each gametocytaemic volunteer by venepuncture. Gametocyte density ranged from 34 to 480/μl of blood (excluding one volunteer who had no microscopically detectable gametocytes but was nevertheless infectious). Blood samples were transferred to pre-warmed membrane feeders (Hemotek Membrane Feeding System, Hemotek Ltd, UK) held at 37.5 °C. On average 217 mosquitoes were used per infective feed (range: 62–799), divided into paper cups with approximately 40 females in each, and allowed to feed through an artificial Parafilm membrane for up to 2 h. In most cases, the blood samples were offered to the mosquitoes within 10 min of being taken, but in three experiments (23, 28 and 29), they were kept for up to 1 ½ h in a water bath at 37 °C before transfer to the membrane feeders. Blood samples from the 42 volunteers were each used in separate experiments except samples from four volunteers (18, 19, 44 and 45), which were used in two insecticide exposure experiments each.

Within 1–3 h, approximately half of the blood-fed mosquitoes were exposed to a net treated with a sub-lethal dose of deltamethrin for 5 min using a wire ball frame, and the other half were exposed to an untreated net as control. After exposure, mosquitoes were kept in paper cups with access to 10 % glucose solution. Temperature and humidity were recorded every 30 min during incubation. Seven days after infection, midguts of surviving females were dissected in 0.25 % mercurochrome in phosphate buffer saline (PBS) solution and examined for oocysts.

### Statistical analysis

Only data for *An. gambiae* s.s. with *kdr-*L1014S homozygous (RR) genotype were included in the statistical analyses to reduce bias due to genetic heterogeneity. Two outcome variables were studied: a) prevalence of oocyst infection and b) intensity of oocyst infection (number of oocysts) among infected mosquitoes. Mantel-Haenszel meta-analysis and forest plot were used with the *metan* procedure in Stata version 13 (StataCorp LP, College Station, Texas 77,845, USA) to study the effect of exposure to sub-lethal doses of deltamethrin on *Plasmodium* infection in *kdr*-resistant *An. gambiae* s.s. mosquitoes, stratifying by feeding experiment. Odds ratios (OR) were calculated to estimate the effect of insecticide exposure on infection prevalence, separately for each gametocyte donor or experiment. Experimental data from different insecticide doses were pooled into two exposure groups: low dose (2.5–5.0 mg/m^2^) and high dose (10.0–16.7 mg/m^2^), as sample sizes for some of the separate doses were inadequate for the analysis. A Mantel-Haenszel pooled OR was calculated as a summary measure of exposure effect across experiments. We used the median of ambient temperature recorded during the experiments (25.3 °C) as cut-off to plot oocyst prevalence charts under low and high temperature conditions.

The effect of deltamethrin exposure was analysed further with multi-level regression models. First, mixed-effects logistic regression was used to study the effect of the insecticide on oocyst infection rate. Secondly, mixed-effects negative binomial regression was used to study the effect of the insecticide on oocyst count in infected mosquitoes.

In both models, the main independent variable was deltamethrin dosage group, as a fixed-effect categorical variable with three levels: control, low dose and high dose, as defined above. In addition, four more fixed-effect explanatory factors were included in both models: the continuous variables average temperature during incubation, gametocyte density, and age of volunteer, and a binary categorical variable indicating whether or not the donor received medication with antimalarials in the previous seven days. To account for the correlation of mosquitoes fed on the same blood sample within each experiment, gametocyte donor volunteers were included as a random (or group) variable. In each model, the two-level random-effects models were compared with models with no random effects using log-likelihood ratio tests to confirm that the mixed-effects models were more appropriate than standard models. The *melogit* and *menbreg* procedures in Stata 13 were used to fit the mixed-effects logistic and mixed-effects negative binomial regressions, respectively.

### Ethics statement

Ethical clearance was obtained from the LSHTM (reference 6454), the Vector Control Division of the Ministry of Health of Uganda (reference VCD-IRC/044), and Uganda National Council of Science and Technology (reference HS 1429). All adult subjects provided written informed consent, and a parent or guardian of any child participant provided written informed consent on their behalf.

## Results

A total of 9502 *An. gambiae* s.l. up to 10 days old were offered an infective blood meal, of which 1285 fully fed. Of these, 935 survived until dissection. Midguts of 862 of the surviving mosquitoes were dissected successfully and examined. Out of these, 763 were identified by PCR as *An. gambiae* s.s. and 73 as *An. arabiensis* (26 mosquitoes could not be identified by PCR). Of the 763 *An. gambiae* s.s., 692 had *kdr-*L1014S homozygous (RR) genotype and were included in the statistical analyses involving effects of deltamethrin on infection.

### Resistance gene frequencies

*An. gambiae* s.s. and *An. arabiensis* were found together in the study area at a ratio of approximately 10 to 1. All but one of the *An. arabiensis* mosquitoes were scored as SS (homozygote susceptible) at the *kdr-*L1014S locus, while in *An. gambiae* s.s. 95 % of the specimens were RR resistant homozygotes (Table 1). All specimens were homozgygous susceptible at the *ace-1* locus.

**Table 1:** Frequencies of *kdr-*L1014S allele in *An. gambiae* s.s. and *An. arabiensis*

*kdr-*L1014S genotype	*An. gambiae* s.s.		*An. arabiensis*	
	*n*	%	*n*	%
SS	4	0.5	72	98.6
RS	34	4.6	0	0.0
RR	694	94.8	1	1.4
Total	732	100	73	100
Allele frequency	97.1 %		1.4 %	

### Mosquito mortality rates

**Fig. 1:**
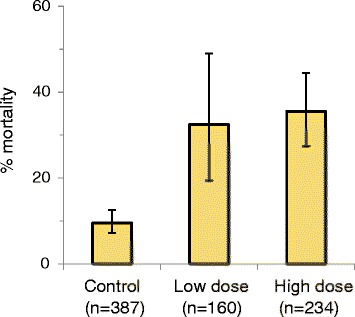
Mortality rates in *kdr-*L1014S resistant homozygous (RR) *An. gambiae* s.s. exposed to different deltamethrin doses: untreated nets (control), and nets treated with low dose (2.5–5.0 mg/m^2^) and high dose (10.0–16.7 mg/m^2^) deltamethrin, assessed after 7 days following exposure for 5 min, at Butemba, Kyankwanzi District, Uganda

The WHO insecticide susceptibility tests confirmed presence of resistance against deltamethrin in the *An. gambiae* s.l. population in the study site, with 71.9 % mortality (*n* = 87). Mortality rates in the mosquitoes fed with infective blood meals were recorded after 7 days of incubation: 9.6, 32.5 and 35.5 % died in the groups exposed to untreated nets, and nets treated with the low-dose and high-dose deltamethrin, respectively (Fig. 1).

### Effect of deltamethrin on infection rate

Forty-one of the 42 volunteers had detectable gametocytemia and one was found to be infectious despite a blood smear showing asexual parasites but no visible gametocytes. The mean age of these volunteers was 17 years (range: 2–56 years). Eight volunteers had taken antimalarial drugs prior to the visit to the health facility; six took artemether-lumefantrine and two took quinine.

**Fig. 2:**
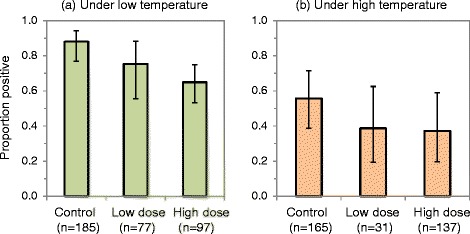
Effects of deltamethrin on *P. falciparum* infection in *kdr* resistant *An. gambiae* s.s. Prevalence rates under (**a)** low temperature (<25.3 °C) and (**b**) high temperature (≥25.3 °C) conditions (control = mosquitoes exposed to untreated nets, low dose = 2.5–5.0 mg/m^2^ deltamethrin and high dose = 10.0–16.7 mg/m^2^ deltamethrin). Mosquitoes were exposed to nets after membrane feeding on blood samples obtained from *P. falciparum* patients (gametocyte donors) at Butemba Health Centre III, Kyankwanzi District, Uganda. Error bars indicate 95 % confidence intervals. Calculations take into account nesting of mosquito samples within gametocyte donor samples

The groups of fed females exposed to deltamethrin had lower infection rates than those exposed to untreated nets. The effect of deltamethrin on infection rates was more pronounced under low temperature conditions (Fig. 2).

**Fig. 3:**
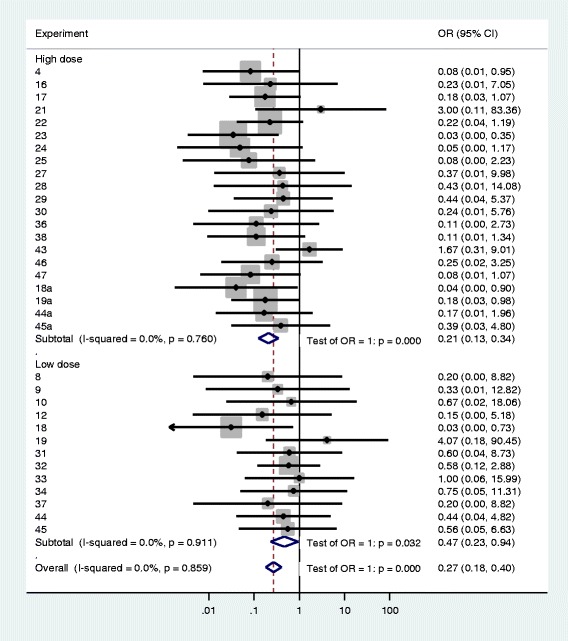
Forest plot of the effects of high and low doses of deltamethrin on *P. falciparum* oocyst infection rates in *kdr-*L1014S resistant *An. gambiae* s.s. The plot shows odds ratio (OR) obtained from meta-analysis of data corresponding to 34 experiments (using blood samples from 30 of the 42 volunteers). Only experiments with sample sizes appropriate for the *metan* procedure calculation were included in the plot (12 experiments had multiple zeros in 2x2 tables and therefore were excluded from the plot). Experiment numbers represent individual volunteers, except when a suffix is used to show more than one experiment per volunteer. For each of the experiments, the OR and 95 % confidence interval (95 % CI) were computed, with OR < 1 indicating lower infection rate of deltamethrin-exposed mosquitoes compared to control. The size of each grey square represents the experiment’s weight and horizontal line indicates 95 % CI. Summary (Mantel-Haenszel pooled) OR estimates for each dose and for all experiments are represented by open diamonds with their lateral tips indicating 95 % confidence limits. The dotted line indicates the overall OR

A meta-analysis forest plot was constructed for 34 experiments to which the *metan* Stata procedure was applicable. The results showed a significant protective effect against infection of both low and high dose exposure to the insecticide (Fig. 3). The Mantel-Haenszel pooled OR was 0.21 for the high dose versus control, 0.47 for the low dose versus control, and 0.27 overall (see Fig. 3 for 95 % CIs). Heterogeneity tests showed a uniform effect across all experiments as indicated by the I^2^ statistic (0.0 % in all cases), which is a measure of the variation of OR attributable to heterogeneity.

Mixed-effects logistic regression analysis, including the data from all experiments using samples from the 42 volunteers, produced very similar estimates of the effect of deltamethrin on infection rates (Table 2). Mosquitoes exposed to the low and high doses had 59 and 80 % lower risk of infection compared to those exposed to untreated nets, and these differences were highly significant (Table 2). This analysis also showed that the mean ambient temperature during the incubation period, which varied between 24.8 and 26.8 °C, had an independent and highly significant effect on risk of infection.

**Table 2:** Mixed-effects logistic regression analysis of *P. falciparum* oocyst prevalence rates

		Odds ratio	Std. Err.	Z	*p*	[95 % Confidence Interval]	
Dose category	Control	1.000	–	–	–	–	–
	Low dose	0.409	0.134	−2.71	0.007	0.215	0.780
	High dose	0.197	0.050	−6.37	<0.001	0.120	0.325
Average temperature (°C)	–	0.179	0.073	−4.23	<0.001	0.081	0.398
Variance of random intercept	–	1.889	0.672	–	–	0.941	3.793

The dependent variable is oocyst infection coded as 0 (negative) and 1 (positive)

Model *χ* ^*2*^_3*d f*_ = 56.63 *p* < 0.001 *n* = 692 number of groups (gametocyte donors) = 42

Gametocyte density, age of gametocyte donor, and prior medication with antimalarial drugs had no statistically significant effects on oocyst infection prevalence (gametocyte density data for one donor was considered an outlier and was excluded from analysis).

### Effect of deltamethrin on oocyst counts

**Fig. 4:**
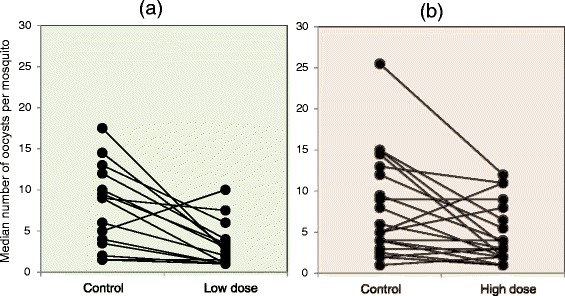
Paired scattergram showing median numbers of oocysts in infected mosquitoes in each experiment for (**a)** the control and low-dose groups, and (**b**) the control and high-dose groups. Each pair of dots connected with a line represents the median numbers in the respective groups in each experiment. Only experiments with median oocyst data for both groups were included in the plot

Oocyst-positive mosquitoes exposed to both the low and high dose deltamethrin had lower infection intensity than positive mosquitoes exposed to untreated nets. The median numbers of oocysts per infected mosquito in each experiment were compared in the control and low-dose groups, and in the control and high-dose groups, using paired scattergrams (Fig. 4). The effect of the insecticide on infection intensity was more pronounced in the low-dose group.

Negative binomial regression of oocyst count of positive mosquitoes showed that exposure to deltamethrin, mean ambient temperature during incubation period and intake of antimalarials in the previous seven days had statistically significant effects (Table 3). Compared with the control group, the number of oocysts per positive mosquito was reduced by 41 and 34 % in the low and high dose groups respectively.

**Table 3:** Mixed-effects negative binomial regression analysis of number of *P. falciparum* oocysts

		Incidence-rate ratio	Std. Err.	Z	*p*	[95 % Confidence Interval]	
Dose category	Control	1.000	–	–	–	–	–
	Low dose	0.586	0.075	−4.18	<0.001	0.456	0.753
	High dose	0.655	0.068	−4.08	<0.001	0.535	0.803
Average temperature (°C)		0.522	0.086	−3.93	<0.001	0.378	0.722
Prior intake of antimalarials	Not taken	1.000	–	–	–	–	–
	Taken	0.595	0.156	−1.98	0.048	0.356	0.995
Ln(alpha^a^)	–	−0.688	0.088	−7.85	<0.001	−0.861	−0.516
Variance of random intercept	–	0.243	0.0899			0.118	0.502

The dependent variable is number of oocysts

Model *χ* ^*2*^_4*d f*_ = 47.72 *p* < 0.001 *n* = 421 number of groups (gametocyte donors) = 40

^a^Alpha = Overdispersion parameter

## Discussion

This study showed that deltamethrin affects development of *P. falciparum* in wild, *kdr-*L1014S resistant *An. gambiae* s.s. in a malaria endemic setting. Exposure to sub-lethal doses of the insecticide shortly after infective feeding reduced both the oocyst prevalence and intensity of infection inside the mosquito.

It is possible that the reduction could be produced by either or both of two possible mechanisms: differential insecticidal killing of infected mosquitoes (as might be seen if infection restored phenotypic susceptibility in genotypically resistant mosquitoes), and/or a direct effect of the insecticide on the parasite inside the mosquito.

Previous laboratory studies have described effects of different pyrethroids on *Plasmodium* sporogony. Deltamethrin was shown to reduce *P. yoelii yoelii* infection rates in *An. stephensi* [14, 16], whereas bioallethrin and fenvalerate affected the parasites at the sporozoite level only [15]. Hill carried out laboratory-based research on the effects of sub-lethal doses of different insecticides on malaria vectors [17]. His findings showed that exposure of insecticide resistant *An. stephensi* to pyrethroids resulted in significant inhibition of *P. yoelii nigeriensis* and *P. falciparum* sporogonic development, whereas no such effect was found with organochlorine, carbamate and organophosphate insecticides. Earlier studies also reported that non-pyrethroid insecticides have no effect on malaria infection in mosquitoes [18, 19]. However, exposure of resistant strains of *An. gambiae* s.s. to bendiocarb and DDT has been shown to reduce *P. falciparum* prevalence [13].

Higher infection rates have been reported in *kdr* resistant mosquitoes compared to susceptible ones although results from different studies were conflicting in terms of the effect on infection intensity at oocyst and sporozoite stages [11, 12]. A recent study showed that *kdr* resistant mosquitoes infected with *P. falciparum* were less able to survive DDT exposure than uninfected mosquitoes during the first seven days post infection, but there was no significant difference in mortality rates between sporozoite-infected and control groups later on [13].

Although *kdr* allele was almost fixed in the *An. gambiae* s.s. population, the susceptibility test data showed relatively high mortality (71.9 %). It is therefore likely that resistance is mediated by a combination of metabolic detoxification mechanisms and *kdr*, and that the resistance phenotype (i.e. strength of expression of resistance) differs between the mosquitoes. Metabolic resistance is a potential confounder in the effect of the insecticide on the parasite as it mediates the amount of insecticide or insecticide metabolites to which the parasite would be exposed. As mosquitoes become resistant and receive sub-lethal doses and survive, the probability of exposure of the parasites to these doses may increase. On the other hand, as detoxification becomes more powerful, most of the insecticide may be metabolised which could mean less exposure of the parasite. Nevertheless, insecticide metabolites or other resistance-related factors could still affect the parasite’s development directly or through their potential effect on the mosquito’s immune system indirectly [25, 26]. Further studies are needed to understand better the potential effects of resistance.

Insecticide dose, mean daily temperature, and medication were all significant variables in our models. The doses of deltamethrin used in this study were much lower than those on a standard long-lasting insecticidal net (LLIN) (e.g. 55 mg/m^2^ in PermaNet® 2.0). Washing and long-term use reduce deltamethrin content of ITNs [24]. So, effects of the kind observed here would be expected not only with new nets, but also older ones—although the effect seems to be dose-dependent, with higher insecticide doses having a bigger impact especially on infection prevalence. However, the reduction of the intensity of infection was more pronounced in the low-dose group than the high-dose group. Some studies have suggested that intensity and prevalence of infection might be regulated by different mechanisms in the mosquito, probably in relation to different immune signalling pathways [27, 28].

We showed that high ambient temperature independently reduced oocyst prevalence and intensity. Temperature affects malaria transmission by affecting the life cycles of both the vector and the parasite. Within the relevant temperature range, sporogony is shorter at higher temperatures [29]. However, higher temperatures have been shown to reduce prevalence of oocyst infection [30], may be detrimental to parasite development [31], and can affect the immune response of mosquitoes [32]. Temperature can also change the effect of insecticides on a mosquito population by modifying mortality rates [33].

Antimalarial medication reduced the intensity of oocyst infections in our study. Six of the volunteers took artemether-lumefantrine and two took quinine within seven days before providing blood samples. These drugs, especially the former, are known to have gametocytocidal properties [34]. In the present study, gametocyte density and age of the donor did not have a significant effect. This could indicate that the insecticide’s effect is probably not at the gametocyte stage.

This study may have implications on the continued use of pyrethroid-based ITNs which have contributed to substantial reduction of malaria mortality in the past decade. As suggested in the study, if pyrethroids affect development of the parasite inside the mosquito, prevention tools dependent on these chemicals will continue to play a major role in malaria control despite vector resistance.

The effect of pyrethroids reported here could explain, at least partly, why resistance has not always led to control failure and ITNs seem to remain effective in most situations [6, 7, 35–37]. More research will be needed to fully understand the mechanisms of interactions between the parasite, different insecticide resistance mechanisms and the insecticide in the mosquito vector, and the roles of these interactions in modulating transmission in the field. Our study suggests that the continued use of pyrethroid treated nets might be helping to prevent failure of malaria control in Africa despite the rapid evolution of insecticide resistance, and supports the efforts to maintain the use of existing effective interventions.

## Conclusions

The use of nets treated with pyrethroid insecticides has contributed to the prevention of millions of deaths due to malaria, but resistance to these insecticides is spreading rapidly in the vector mosquitoes in Africa. We investigated whether the chemicals could affect malaria parasites inside resistant mosquitoes in an endemic area. The study showed that, with locally-sampled *P. falciparum* parasites and *An. gambiae* s.s., doses of pyrethroids that are sub-lethal for resistant mosquitoes can interfere with parasite development inside mosquitoes, significantly reducing both the proportion of infected mosquitoes and the intensity of infection. This mechanism could enable pyrethroid-treated nets to prevent malaria transmission despite increasing vector resistance.
